# Validity of Self-reported Participation in Cancer Screenings and Health Checkups in Japan

**DOI:** 10.2188/jea.JE20240090

**Published:** 2025-01-05

**Authors:** Isao Muraki, Tomotaka Sobue, Kazumasa Yamagishi, Shoichiro Tsugane, Norie Sawada, Hiroyasu Iso

**Affiliations:** 1 Public Health, Department of Social Medicine, Osaka University Graduate School of Medicine, Osaka, Japan; 2 Environmental Medicine, Department of Social Medicine, Osaka University Graduate School of Medicine, Osaka, Japan; 3 Department of Public Health, Graduate School of Medicine, Juntendo University, Tokyo, Japan; 4 Department of Public Health Medicine, Institute of Medicine, and Health Services Research and Development Center, University of Tsukuba, Ibaraki, Japan; 5 International University of Health and Welfare Graduate School of Public Health, Tokyo, Japan; 6 Division of Cohort Research, National Cancer Center Institute for Cancer Control, Tokyo, Japan; 7 Institute for Global Health Policy Research Center, Bureau of International Health Cooperation, National Center for Global Health and Medicine, Tokyo, Japan

**Keywords:** self-reported participation, cancer screening, health checkups, validation study

## Abstract

**Background:**

The participation rate for screening is regarded as a useful indicator for preventing cancer and cardio-metabolic disease. However, the validity of self-reported screening participation has not yet been thoroughly evaluated in Japan. We aimed to examine its validity using the municipal screening records among the Japanese population.

**Methods:**

We included 3,060 men and 3,860 women insured by the National Health Insurance for residents aged <75 years or the Medical Care System for the Elderly aged ≥75 years in the Chikusei area of the Japan Public Health Center-based Prospective Study for the Next Generation. They were asked about their participation in cancer screenings and health checkups during the previous year. We compared their responses to the municipal records and calculated the sensitivity and specificity of self-reported screening participation.

**Results:**

The sensitivity and specificity of self-reported participation were 0.49 and 0.86 for lung cancer screening, 0.67 and 0.85 for colorectal cancer screening, 0.77 and 0.79 for stomach cancer screening, and 0.86 and 0.65 for health checkup, respectively. Among women, the sensitivity and specificity were 0.83 and 0.81 for breast cancer and 0.85 and 0.90 for cervical cancer, respectively.

**Conclusion:**

Self-reported cancer screening participation for colorectal, stomach, breast, and cervical cancers had moderate-to-high sensitivity and specificity. Self-reported participation, especially for lung cancer screening and health checkups, should be carefully interpreted when assessing the performance of preventive measures.

## INTRODUCTION

Organized cancer screening is an efficient measure to reduce mortality from colorectal,^1^ breast,^2^ and cervical cancers.^3^ To enhance these preventive effects, participation in cancer screening has been encouraged for individuals who can obtain more benefits from screening than for those who may suffer from overdiagnosis and excessive treatment.^4^^–^^7^ Monitoring participation in cancer screening is important for organized cancer screening practices. In Organisation for Economic Co-operation and Development countries, participation in cancer screening is measured using program-based data in two-thirds to three-fourths of countries and using self-reporting in the rest, including Japan.^8^^,^^9^ The validity of self-reported participation ranged from 0.32 to 0.98 in sensitivity and 0.58 to 0.93 in specificity for the fecal occult blood test (FOBT),^10^ 0.78 to 1.00 and 0.20 to 0.94 for mammography, and 0.82 to 0.99 and 0.15 to 0.75 for Pap smears, respectively.^11^ These variations in sensitivity and specificity across the study populations may be due to differences in their historical backgrounds and participation rates of screening.^10^^,^^11^ Furthermore, information on the validity of self-reported participation in lung and stomach cancer screenings and health checkups has been scarce because they are not commonly conducted as organized cancer screening.^12^^,^^13^ In this study, we aimed to examine the sensitivity and specificity of self-reported screening participation compared to the municipal records of cancer screenings and health checkups among Japanese.

## METHODS

### Study design and population

Using a cross-sectional study design, we compared self-reported screening participation obtained in the Japan Public Health Center-based Prospective Study for the Next Generation (JPHC-NEXT) with the actual screening participation ascertained by the municipal screening records. We first identified the JPHC-NEXT ID of participants in the municipal screening records using person-identifiable information, such as sex, birth date, and name. Then, we merged the two datasets by the JPHC-NEXT ID. The JPHC-NEXT study started in the 2011–2016 fiscal years (from April 2011 to March 2017) with the enrollment of 115,385 individuals aged 40–74 years from seven prefectural areas.^14^ All participants completed a self-administered questionnaire on lifestyle, dietary intake, and medical history from the 2011–2016 fiscal year and subsequently every about 5 years thereafter. For the current analysis, we obtained individual data on participation in cancer screenings and health checkups from April 1st, 2015 to March 31st, 2020 in Chikusei City, an area of the JPHC-NEXT study. Chikusei City is a rural plain area in central east Japan, and the population was 104,573 men and women (proportion aged ≥65 years: 28.0%) on October 1st, 2015. In that city, the National Health Insurance covered one-third of the population, and the Medical Care System for the Elderly aged ≥75 years covered one-eighth of the population.

Of 17,106 participants in the JPHC-NEXT study of the Chikusei cohort, we included 6,923 participants insured continuously for at least 2 years before the response date for the 2016–2020 survey by the National Health Insurance for residents aged <75 years or the Medical Care System for the Elderly aged ≥75 years because biennial screening for stomach, breast, and cervical cancers is recommended in Japan. We excluded those with erroneous responses, such as women participating in prostate cancer screening (*n* = 3) and those with missing data on self-reported participation in cancer screenings or health checkups at the 2016–2020 survey (*n* = 119). Finally, we included 6,920 participants aged 44–80 years.

All participants were informed about the study protocol and provided written informed consent to participate in the JPHC-NEXT study. The JPHC-NEXT study was approved by the Institutional Ethics Committees of the National Cancer Center and Osaka University.

### Self-reported participation in cancer screenings and health checkups

Participation in cancer screenings and health checkups was asked ‘Did you have any health and cancer screenings in the last year?’ Responding ‘yes’ to the first question, participants chose the types of cancer screenings and health checkups in which they participated in the last year from the following eight items: ‘regular health checkup (not including cancer screening only)’, ‘stomach cancer screening’, ‘lung cancer screening’, ‘colorectal cancer screening’, ‘cervical cancer screening’, ‘breast cancer screening’, ‘prostate cancer screening’, and ‘screening for other cancers’. We did not provide additional information on individual cancer screenings and health checkups such as, for example, listing enhanced gastric radiography or gastric endoscopy for stomach cancer screening. The Japanese version of the 5-year follow-up questionnaire was available at the JPHC-NEXT study website (https://epi.ncc.go.jp/jphcnext/documents/index.html).

### The reference standards of participation in cancer screenings and health checkups

We used the municipal data from the screening for stomach, lung, colorectal, breast, and cervical cancers and health checkups for the assessment of cardiovascular risk factors as the reference standards for a history of participation in cancer screenings and health checkups. The Japanese screening system is summarized in eMaterial 1.

### Statistical analysis

We calculated the participation rate and three validity indicators: sensitivity, specificity, and concordance rate. Their calculation methods were described in detail in eMaterial 1. We used arbitrary benchmarks of indicators of ≥0.90 as very high, 0.80–0.89 as high, 0.65–0.79 as moderate, and <0.65 as low, as recently reported,^15^ albeit with no standard criteria. In the primary analysis, we compared the responses to the questionnaire with the screening participation records during the last fiscal year (Scenario 1). To assess the differences in the validity of the self-reported history of screening participation among different populations, we also calculated the validity indicators stratified by age, sex, and level of education. To assess the impact of interpretation error in the targeted interval or recall error, we created three modified scenarios to calculate the above indicators of self-reported screening participation compared with the municipal records. We compared the responses to the questionnaire with the screening records within 12 months before the responding month (Scenario 2). For screening of stomach, breast, and cervical cancers that are recommended biennially,^16^ we compared the responses to the questionnaire with the screening participation record within the last 2 fiscal years (Scenario 3), and within 24 months before the responding month (Scenario 4) to examine the impact of authorized recommendation. To keep the indicators comparable between scenarios, we used the data in the 2017–2019 survey available for all scenarios.

All analyses were performed using SAS 9.4 (SAS Institute Inc., Cary, NC, USA).

## RESULTS

At response to the questionnaire, over half of the current study population were aged ≥70 years, female, unemployed, and had high school-level education and a family history of cancer (Table 1). More than four-fifths of the participants were married. Half or more participants had lung cancer screening and health checkups. Around two-fifths of participants had colorectal cancer screening. Around one-third or fewer participants had screening for stomach, breast, and cervical cancers.

**Table 1.** Characteristics of study participants at 5-year follow-up survey in the JPHC-NEXT study

	*N*	%
Total	6,920	
Age		
44–49 years	271	(3.9)
50–59 years	565	(8.2)
60–69 years	2,412	(34.9)
70–80 years	3,672	(53.1)
Male	3,060	(44.2)
Marital status		
Married	5,257	(76.0)
Not married	1,466	(21.2)
Missing	197	(2.9)
Education		
Junior high school	1,729	(25.0)
High school	3,624	(52.4)
College or higher	1,480	(21.4)
Missing	87	(1.3)
Occupational status, %		
Not employed	3,733	(54.0)
Self-employed	1,994	(28.8)
Employed	1,063	(15.4)
Missing	130	(1.9)
Income level, %		
<3,000,000 yen	3,062	(44.3)
3,000,000 to <6,000,000 yen	2,261	(32.7)
6,000,000 to <9,000,000 yen	537	(7.8)
9,000,000 yen or more	430	(6.2)
Missing	630	(9.1)
Smoking status, %		
Never smokers	4,059	(58.7)
Former smokers	2,120	(30.6)
Current smokers	712	(10.3)
Missing	29	(0.4)
Alcohol drinking status, %		
Never drinkers	3,080	(44.5)
Former drinkers	367	(5.3)
Current drinkers	3,429	(49.6)
Missing	44	(0.6)
Family history of cancer		
No	3,351	(48.4)
Yes	3,569	(51.6)
Any cancer history		
No	6,077	(87.8)
Yes	843	(12.2)

The sensitivity, specificity, and concordance rate of the self-reported history for cancer screenings and health checkups are shown in Table 2, Table 3, Table 4, Table 5, Table 6, and Table 7. Participation in lung cancer screening with chest radiography was largely underreported in examinees (sensitivity = 0.49) and moderately overreported in non-examinees (specificity = 0.86), resulting in a low agreement between self-report and municipal records (concordance = 0.65) (Table 2). These results were generally similar between men and women and among educational levels. Participation in colorectal cancer screening with an FOBT was moderately underreported in examinees (sensitivity = 0.67) and moderately overreported in non-examinees (specificity = 0.85), resulting in moderate agreement (concordance = 0.77) (Table 3). Overreporting of colorectal cancer screening participation was more extensive amongst older people. Participation in stomach cancer screening with enhanced gastric radiography was slightly under-reported in examinees (sensitivity = 0.77) and moderately over-reported in non-examinees (specificity = 0.79) (Table 4). The overall agreement was moderate between self-report and municipal records of stomach cancer screening (concordance = 0.79). Participation in health checkups was almost accurately reported in examinees (sensitivity = 0.86) but largely overreported in non-examinees (specificity = 0.65), resulting in moderate agreement (concordance = 0.78) (Table 5).

**Table 2.** Validity indicators of self-reported history of lung cancer screening in men and women

	Number of participants					Participation rate, % (95% CI)	Sensitivity (95% CI)	Specificity (95% CI)	Concordance rate (95% CI)

	Total	TP	FN	FP	TN				
Lung cancer									
Total	6,920	1,920	2,026	410	2,564	57.0 (55.9–58.2)	0.49 (0.47–0.50)	0.86 (0.85–0.87)	0.65 (0.64–0.66)
Men	3,060	837	838	173	1,212	54.7 (53.0–56.5)	0.50 (0.48–0.52)	0.88 (0.86–0.89)	0.67 (0.65–0.69)
Women	3,860	1,083	1,188	237	1,352	58.8 (57.3–60.4)	0.48 (0.46–0.49)	0.85 (0.84–0.86)	0.63 (0.62–0.65)
44–49 years	271	84	73	10	104	57.9 (52.1–63.8)	0.54 (0.48–0.59)	0.91 (0.88–0.95)	0.69 (0.64–0.75)
50–59 years	565	166	160	18	221	57.7 (53.6–61.8)	0.51 (0.47–0.55)	0.92 (0.90–0.95)	0.68 (0.65–0.72)
60–69 years	2,412	722	772	156	762	61.9 (60.0–63.9)	0.48 (0.46–0.50)	0.83 (0.82–0.85)	0.62 (0.60–0.63)
70–80 years	3,672	948	1,021	226	1,477	53.6 (52.0–55.2)	0.48 (0.47–0.50)	0.87 (0.86–0.88)	0.66 (0.65–0.68)
Junior high school	1,729	429	508	82	710	54.2 (51.8–56.5)	0.46 (0.43–0.48)	0.90 (0.88–0.91)	0.66 (0.64–0.68)
High school	3,624	1,075	1,112	211	1,226	60.3 (58.8–61.9)	0.49 (0.48–0.51)	0.85 (0.84–0.86)	0.63 (0.62–0.65)
College or higher	1,480	395	382	110	593	52.5 (50.0–55.0)	0.51 (0.48–0.53)	0.84 (0.83–0.86)	0.67 (0.64–0.69)
Not employed	3,733	1,007	1,027	238	1,461	54.5 (52.9–56.1)	0.50 (0.48–0.51)	0.86 (0.85–0.87)	0.66 (0.65–0.68)
Self-employed	1,994	606	642	105	641	62.6 (60.5–64.7)	0.49 (0.46–0.51)	0.86 (0.84–0.87)	0.63 (0.60–0.65)
Employed	1,063	274	316	62	411	55.5 (52.5–58.5)	0.46 (0.43–0.49)	0.87 (0.85–0.89)	0.64 (0.62–0.67)
Having a family history of cancer	3,569	1,056	1,008	235	1,270	57.8 (56.2–59.5)	0.51 (0.50–0.53)	0.84 (0.83–0.86)	0.65 (0.64–0.67)
Having any cancer history	843	181	187	70	405	43.7 (40.3–47.0)	0.49 (0.46–0.53)	0.85 (0.83–0.88)	0.70 (0.66–0.73)

CI, confidence interval; FN, false negative; FP, false positive; TN, true negative; TP, true positive.

**Table 3.** Validity indicators of self-reported history of colorectal cancer screening in men and women

	Number of participants					Participation rate, % (95% CI)	Sensitivity (95% CI)	Specificity (95% CI)	Concordance rate (95% CI)

	Total	TP	FN	FP	TN				
Colorectal cancer									
Total	6,920	2,077	1,024	591	3,228	44.8 (43.6–46.0)	0.67 (0.66–0.68)	0.85 (0.84–0.85)	0.77 (0.76–0.78)
Men	3,060	837	457	288	1,478	42.3 (40.5–44.0)	0.65 (0.63–0.66)	0.84 (0.82–0.85)	0.76 (0.74–0.77)
Women	3,860	1,240	567	303	1,750	46.8 (45.2–48.4)	0.69 (0.67–0.70)	0.85 (0.84–0.86)	0.77 (0.76–0.79)
44–49 years	271	80	35	10	146	42.4 (36.6–48.3)	0.70 (0.64–0.75)	0.94 (0.91–0.97)	0.83 (0.79–0.88)
50–59 years	565	167	75	23	300	42.8 (38.8–46.9)	0.69 (0.65–0.73)	0.93 (0.91–0.95)	0.83 (0.80–0.86)
60–69 years	2,412	786	390	218	1,018	48.8 (46.8–50.8)	0.67 (0.65–0.69)	0.82 (0.81–0.84)	0.75 (0.73–0.77)
70–80 years	3,672	1,044	524	340	1,764	42.7 (41.1–44.3)	0.67 (0.65–0.68)	0.84 (0.83–0.85)	0.76 (0.75–0.78)
Junior high school	1,729	459	249	135	886	40.9 (38.6–43.3)	0.65 (0.63–0.67)	0.87 (0.85–0.88)	0.78 (0.76–0.80)
High school	3,624	1,161	566	293	1,604	47.7 (46.0–49.3)	0.67 (0.66–0.69)	0.85 (0.83–0.86)	0.76 (0.75–0.78)
College or higher	1,480	438	197	160	685	42.9 (40.4–45.4)	0.69 (0.67–0.71)	0.81 (0.79–0.83)	0.76 (0.74–0.78)
Not employed	3,733	1,125	525	328	1,755	44.2 (42.6–45.8)	0.68 (0.67–0.70)	0.84 (0.83–0.85)	0.77 (0.76–0.78)
Self-employed	1,994	600	339	168	887	47.1 (44.9–49.3)	0.64 (0.62–0.66)	0.84 (0.82–0.86)	0.75 (0.73–0.76)
Employed	1,063	318	133	83	529	42.4 (39.5–45.4)	0.71 (0.68–0.73)	0.86 (0.84–0.88)	0.80 (0.77–0.82)
Having a family history of cancer	3,569	1,157	542	341	1,529	47.6 (46.0–49.2)	0.68 (0.67–0.70)	0.82 (0.80–0.83)	0.75 (0.74–0.77)
Having any cancer history	843	191	128	105	419	37.8 (34.6–41.1)	0.60 (0.57–0.63)	0.80 (0.77–0.83)	0.72 (0.69–0.75)

CI, confidence interval; FN, false negative; FP, false positive; TN, true negative; TP, true positive.

**Table 4.** Validity indicators of self-reported history of stomach cancer screening in men and women

	Number of participants					Participation rate, % (95% CI)	Sensitivity (95% CI)	Specificity (95% CI)	Concordance rate (95% CI)

	Total	TP	FN	FP	TN				
Stomach cancer									
Total	6,920	910	279	1,175	4,556	17.2 (16.3–18.1)	0.77 (0.76–0.78)	0.79 (0.79–0.80)	0.79 (0.78–0.80)
Men	3,060	457	165	446	1,992	20.3 (18.9–21.8)	0.73 (0.72–0.75)	0.82 (0.80–0.83)	0.80 (0.79–0.81)
Women	3,860	453	114	729	2,564	14.7 (13.6–15.8)	0.80 (0.79–0.81)	0.78 (0.77–0.79)	0.78 (0.77–0.79)
44–49 years	271	48	14	16	193	22.9 (17.9–27.9)	0.77 (0.72–0.82)	0.92 (0.89–0.96)	0.89 (0.85–0.93)
50–59 years	565	98	28	35	404	22.3 (18.9–25.7)	0.78 (0.74–0.81)	0.92 (0.90–0.94)	0.89 (0.86–0.91)
60–69 years	2,412	338	95	393	1,586	18.0 (16.4–19.5)	0.78 (0.76–0.80)	0.80 (0.79–0.82)	0.80 (0.78–0.81)
70–80 years	3,672	426	142	731	2,373	15.5 (14.3–16.6)	0.75 (0.74–0.76)	0.76 (0.75–0.78)	0.76 (0.75–0.78)
Junior high school	1,729	170	57	309	1,193	13.1 (11.5–14.7)	0.75 (0.73–0.77)	0.79 (0.78–0.81)	0.79 (0.77–0.81)
High school	3,624	514	153	589	2,368	18.4 (17.1–19.7)	0.77 (0.76–0.78)	0.80 (0.79–0.81)	0.80 (0.78–0.81)
College or higher	1,480	220	62	264	934	19.1 (17.1–21.1)	0.78 (0.76–0.80)	0.78 (0.76–0.80)	0.78 (0.76–0.80)
Not employed	3,733	440	141	710	2,442	15.6 (14.4–16.7)	0.76 (0.74–0.77)	0.77 (0.76–0.79)	0.77 (0.76–0.79)
Self-employed	1,994	305	92	312	1,285	19.9 (18.2–21.7)	0.77 (0.75–0.79)	0.80 (0.79–0.82)	0.80 (0.78–0.82)
Employed	1,063	154	39	133	737	18.2 (15.8–20.5)	0.80 (0.77–0.82)	0.85 (0.83–0.87)	0.84 (0.82–0.86)
Having a family history of cancer	3,569	512	138	669	2,250	18.2 (16.9–19.5)	0.79 (0.77–0.80)	0.77 (0.76–0.78)	0.77 (0.76–0.79)
Having any cancer history	843	57	22	201	563	9.4 (7.4–11.3)	0.72 (0.69–0.75)	0.74 (0.71–0.77)	0.74 (0.71–0.77)

CI, confidence interval; FN, false negative; FP, false positive; TN, true negative; TP, true positive.

**Table 5.** Validity indicators of self-reported history of health checkups in men and women

	Number of participants					Participation rate, % (95% CI)	Sensitivity (95% CI)	Specificity (95% CI)	Concordance rate (95% CI)

	Total	TP	FN	FP	TN				
Health checkups									
Total	6,920	3,704	593	923	1,700	62.1 (61.0–63.2)	0.86 (0.85–0.87)	0.65 (0.64–0.66)	0.78 (0.77–0.79)
Men	3,060	1,581	262	436	781	60.2 (58.5–62.0)	0.86 (0.85–0.87)	0.64 (0.62–0.66)	0.77 (0.76–0.79)
Women	3,860	2,123	331	487	919	63.6 (62.1–65.1)	0.87 (0.85–0.88)	0.65 (0.64–0.67)	0.79 (0.78–0.80)
44–49 years	271	129	35	32	75	60.5 (54.7–66.3)	0.79 (0.74–0.84)	0.70 (0.65–0.76)	0.75 (0.70–0.80)
50–59 years	565	299	46	56	164	61.1 (57.0–65.1)	0.87 (0.84–0.89)	0.75 (0.71–0.78)	0.82 (0.79–0.85)
60–69 years	2,412	1,460	196	295	461	68.7 (66.8–70.5)	0.88 (0.87–0.89)	0.61 (0.59–0.63)	0.80 (0.78–0.81)
70–80 years	3,672	1,816	316	540	1,000	58.1 (56.5–59.7)	0.85 (0.84–0.86)	0.65 (0.63–0.66)	0.77 (0.75–0.78)
Junior high school	1,729	777	179	257	516	55.3 (52.9–57.6)	0.81 (0.79–0.83)	0.67 (0.65–0.69)	0.75 (0.73–0.77)
High school	3,624	2,071	296	449	808	65.3 (63.8–66.9)	0.87 (0.86–0.89)	0.64 (0.63–0.66)	0.79 (0.78–0.81)
College or higher	1,480	821	103	205	351	62.4 (60.0–64.9)	0.89 (0.87–0.90)	0.63 (0.61–0.66)	0.79 (0.77–0.81)
Not employed	3,733	1,974	280	479	1,000	60.4 (58.8–61.9)	0.88 (0.87–0.89)	0.68 (0.66–0.69)	0.80 (0.78–0.81)
Self-employed	1,994	1,128	204	238	424	66.8 (64.7–68.9)	0.85 (0.83–0.86)	0.64 (0.62–0.66)	0.78 (0.76–0.80)
Employed	1,063	543	89	196	235	59.5 (56.5–62.4)	0.86 (0.84–0.88)	0.55 (0.52–0.58)	0.73 (0.71–0.76)
Having a family history of cancer	3,569	1,992	274	479	824	63.5 (61.9–65.1)	0.88 (0.87–0.89)	0.63 (0.62–0.65)	0.79 (0.78–0.80)
Having any cancer history	843	375	56	119	293	51.1 (47.8–54.5)	0.87 (0.85–0.89)	0.71 (0.68–0.74)	0.79 (0.77–0.82)

CI, confidence interval; FN, false negative; FP, false positive; TN, true negative; TP, true positive.

**Table 6.** Validity indicators of self-reported history of breast cancer screenings among women

	Number of participants					Participation rate, % (95% CI)	Sensitivity (95% CI)	Specificity (95% CI)	Concordance rate (95% CI)

	Total	TP	FN	FP	TN				
Breast cancer									
Total	3,860	839	169	542	2,310	26.1 (24.7–27.5)	0.83 (0.82–0.84)	0.81 (0.80–0.82)	0.82 (0.80–0.83)
44–49 years	130	29	4	29	68	25.4 (17.9–32.9)	0.88 (0.82–0.93)	0.70 (0.62–0.78)	0.75 (0.67–0.82)
50–59 years	301	67	12	44	178	26.2 (21.3–31.2)	0.85 (0.81–0.89)	0.80 (0.76–0.85)	0.81 (0.77–0.86)
60–69 years	1,436	365	77	242	752	30.8 (28.4–33.2)	0.83 (0.81–0.85)	0.76 (0.73–0.78)	0.78 (0.76–0.80)
70–80 years	1,993	378	76	227	1,312	22.8 (20.9–24.6)	0.83 (0.82–0.85)	0.85 (0.84–0.87)	0.85 (0.83–0.86)
Junior high school	959	178	43	93	645	23.0 (20.4–25.7)	0.81 (0.78–0.83)	0.87 (0.85–0.89)	0.86 (0.84–0.88)
High school	2,074	490	96	290	1,198	28.3 (26.3–30.2)	0.84 (0.82–0.85)	0.81 (0.79–0.82)	0.81 (0.80–0.83)
College or higher	781	164	29	149	439	24.7 (21.7–27.7)	0.85 (0.82–0.87)	0.75 (0.72–0.78)	0.77 (0.74–0.80)
Not employed	2,357	497	102	334	1,424	25.4 (23.7–27.2)	0.83 (0.81–0.84)	0.81 (0.79–0.83)	0.82 (0.80–0.83)
Self-employed	836	195	40	121	480	28.1 (25.1–31.2)	0.83 (0.80–0.86)	0.80 (0.77–0.83)	0.81 (0.78–0.83)
Employed	596	135	25	78	358	26.8 (23.3–30.4)	0.84 (0.81–0.87)	0.82 (0.79–0.85)	0.83 (0.80–0.86)
Having a family history of cancer	2,072	495	84	319	1,174	27.9 (26.0–29.9)	0.85 (0.84–0.87)	0.79 (0.77–0.80)	0.81 (0.79–0.82)
Having any cancer history	419	52	7	116	244	14.1 (10.8–17.4)	0.88 (0.85–0.91)	0.68 (0.63–0.72)	0.71 (0.66–0.75)

CI, confidence interval; FN, false negative; FP, false positive; TN, true negative; TP, true positive.

**Table 7.** Validity indicators of self-reported history of cervical cancer screenings among women

	Number of participants					Participation rate, % (95% CI)	Sensitivity (95% CI)	Specificity (95% CI)	Concordance rate (95% CI)

	Total	TP	FN	FP	TN				
Cervical cancer									
Total	3,860	955	175	281	2,449	29.3 (27.8–30.7)	0.85 (0.83–0.86)	0.90 (0.89–0.91)	0.88 (0.87–0.89)
44–49 years	130	41	5	9	75	35.4 (27.2–43.6)	0.89 (0.84–0.94)	0.89 (0.84–0.95)	0.89 (0.84–0.95)
50–59 years	301	87	15	26	173	33.9 (28.5–39.2)	0.85 (0.81–0.89)	0.87 (0.83–0.91)	0.86 (0.83–0.90)
60–69 years	1,436	432	78	114	812	35.5 (33.0–38.0)	0.85 (0.83–0.87)	0.88 (0.86–0.89)	0.87 (0.85–0.88)
70–80 years	1,993	395	77	132	1,389	23.7 (21.8–25.5)	0.84 (0.82–0.85)	0.91 (0.90–0.93)	0.90 (0.88–0.91)
Junior high school	959	175	39	57	688	22.3 (19.7–25.0)	0.82 (0.79–0.84)	0.92 (0.91–0.94)	0.90 (0.88–0.92)
High school	2,074	563	104	133	1,274	32.2 (30.1–34.2)	0.84 (0.83–0.86)	0.91 (0.89–0.92)	0.89 (0.87–0.90)
College or higher	781	212	30	87	452	31.0 (27.7–34.2)	0.88 (0.85–0.90)	0.84 (0.81–0.86)	0.85 (0.83–0.88)
Not employed	2,357	556	104	173	1,524	28.0 (26.2–29.8)	0.84 (0.83–0.86)	0.90 (0.89–0.91)	0.88 (0.87–0.90)
Self-employed	836	232	39	67	498	32.4 (29.2–35.6)	0.86 (0.83–0.88)	0.88 (0.86–0.90)	0.87 (0.85–0.90)
Employed	596	152	29	35	380	30.4 (26.7–34.1)	0.84 (0.81–0.87)	0.92 (0.89–0.94)	0.89 (0.87–0.92)
Having a family history of cancer	2,072	565	88	158	1,261	31.5 (29.5–33.5)	0.87 (0.85–0.88)	0.89 (0.88–0.90)	0.88 (0.87–0.90)
Having any cancer history	419	78	13	45	283	21.7 (17.8–25.7)	0.86 (0.82–0.89)	0.86 (0.83–0.90)	0.86 (0.83–0.89)

CI, confidence interval; FN, false negative; FP, false positive; TN, true negative; TP, true positive.

Regarding female cancer screening, participation in breast cancer screening with mammography or breast echography was slightly underreported in examinees (sensitivity = 0.83) and slightly overreported in non-examinees (specificity = 0.81), resulting in moderate agreement (concordance = 0.78) (Table 6). Overreporting of participation in breast cancer screening was greater in younger women and women with higher education levels. Participation in cervical cancer screening with Pap smears was slightly misreported in both examinees and non-examinees (sensitivity = 0.85, and specificity = 0.90), leading to high agreement between self-report and municipal records (concordance = 0.88) (Table 7). Underreporting of participation in cervical cancer screening was more likely to occur among older examinees. Overreporting in non-examinees was greater in women with lower educational levels.

To estimate the impact of errors in interpreting the targeted interval for previous screening participation, we calculated the validity indicators according to four possible scenarios. No material differences in validity were found among the four scenarios, suggesting that no systematic bias was caused by interpretation or recall error (eTable 1).

## DISCUSSION

We confirmed the moderate-to-high validity of the self-reported screening participation for colorectal, stomach, and cervical cancers. Underreporting was observed for lung cancer screening, while overreporting was observed for health checkups. The validity of self-reported screening histories did not vary substantially by age, sex, education level, occupation status, family history of cancer, and participant’s history of cancer. To the best of our knowledge, no validation study has been conducted regarding self-reported participation in health checkups.

The validity of self-reported screening history has been well examined for colorectal cancer using FOBT, breast cancer using mammography or breast echography, and cervical cancer using Pap smears. In the meta-analyses of validation studies from Western countries, the pooled sensitivity and specificity of cancer screening participation were 78% and 80% for FOBT,^10^ 95% and 64% for mammography, and 95% and 47% for Pap smears, respectively.^11^ In our study, the sensitivity of the self-reported history of FOBT, mammography/breast echography, and Pap smears was slightly lower, whereas the specificity for mammography/breast echography and Pap smears was higher than in previous studies in Western countries.^10^^,^^11^ It is frequently observed that the sensitivity becomes lower when the prevalence is lower, and the specificity becomes lower when the prevalence is higher.^10^^,^^17^^–^^19^ Therefore, the difference in the validity of self-reported screening histories for breast and cervical cancers can be mostly explained by the lower screening rates in Japan than in Western countries.^8^^,^^9^

The self-reported history of lung and stomach cancer screenings and health checkups has not been well validated.^20^^,^^21^ In a United States study, the sensitivity and specificity of chest radiography participation, not being asked as lung cancer screening participation, were 0.84 and 0.28, respectively.^20^ One possible reason for the underreporting of lung cancer screening in the present study may be misrecognition due to the long history of regular tuberculosis screening using chest radiography in Japan from 1951 through 2004.^22^ The memory of regular tuberculosis screening using chest radiography may cause some residents to misrecognize their chest radiography as a tuberculosis screening, even though chest radiography is explained as lung cancer screening in Chikusei City. This explanation is supported by the slightly better validity of self-reported participation in lung cancer screening among younger individuals in the present study.

A validation study of the self-reported screening history of enhanced gastric radiography was conducted in a Japanese population.^21^ The sensitivity and specificity of the 5-year cumulative screening were 100% and 75.6%, respectively. Multiple screening opportunities over a long period could increase the screening participation rate and result in high sensitivity.

The strength of the present study was its large sample size, which helped to narrow the confidence interval. However, this study has some limitations. First, the informal screening participation at the participants’ own expense was not included in the reference standards due to no availability in the municipal registry. Second, the accuracy of self-reported screening participation may be overestimated due to the higher health awareness and literacy in our study population than in the general Japanese population. The participation rates for all cancer screenings and health checkups were higher in the current study than in the national statistics under the National Health Insurance.^23^^,^^24^ Finally, our findings may only be generalized to Japanese insured by the National Health Insurance or the Medical Insurance System for the Elderly aged ≥75 years, but not to employees covered by the Employee’s Health Insurance. Since employees have at least two cancer screening opportunities at a municipality and workplaces, it is hard to evaluate the validity of self-report participation in cancer screenings among them. However, evidence on the validity of self-reported participation in lung and stomach cancer screenings and health checkups has been scarce to date and can be useful for policymakers in other countries to consider these screenings.

In conclusion, most self-reports of participation in cancer screening have moderate-to-high sensitivity and specificity. However, lung cancer screening participation was underreported, while health checkup participation was overreported. Therefore, self-reported participation, especially for lung cancer screening and health checkups, should be carefully interpreted when assessing the performance of preventive measures.

## SUPPLEMENTARY MATERIAL

eMaterial 1. Screening system in Japan

eTable 1. Validity indicators of self-reported history of cancer screenings and health checkups according to different scenarios using data in the 2017–2019 survey
