# Supplementary material for: Validity of Self-reported Participation in Cancer Screenings and Health Checkups in Japan
Source: J Epidemiol. 2025 Jan 5;35(1):47–52. doi: 10.2188/jea.JE20240090 (PMC11637815; doi:10.2188/jea.JE20240090)
Supplement: Supplementary file 1 [file je-35-047-s001.pdf]

eMaterial 1. Screening system in Japan

Screening for stomach, lung, colorectal, breast, and cervical cancers was provided at the municipality as part of governmental preventive measures for cancer according to the Health and Medical Service Act for the Aged in 1983–2007 and the Health Promotion Act since 2008.[1] Chikusei City provided chest radiography for lung cancer screening, enhanced gastric radiography for stomach cancer screening, the fecal occult blood test for colorectal cancer screening, for men and women aged ≥40 years; mammography or breast echography for breast cancer screening for women aged ≥40 years; and cervical cytology (Pap smears) for cervical cancer screening for those aged ≥20 years. Annual opportunities for health checkups (generally named 'Specific Health Checkups') have been provided to the insured aged 40–74 years in municipalities by healthcare insurers since 2008 according to the Act on Assurance of Medical Care for the Elderly in Japan.[2] Individuals aged 75 years or older have annual opportunities for health checkups by the prefecture-level Association of Medical Care System for Older People.[2]

Calculation of validity indicators

We counted the number of participants reporting their screening participation and confirmed with screening records (true positive, TP), those reporting their screening participation but not confirmed (false positive, FP), those not reporting their screening participation but existing their screening participation record (false negative, FN), and those not reporting their screening participation and confirmed their non-participation with screening records (true negative, TN). The screening participation rate was

calculated as the sum of TP and FN divided by the number of subjects (N). The sensitivity (Sens. = TP / (TP + FN)), specificity (Spec. = TN / (FP + TN)), and concordance rate (Conc. = (TP + TN) / N) as valid indicators of a self-reported history of screening participation. A two-sided 95% confidence interval of the proportion was calculated as a point estimate plus or minus the square root of the proportion multiplied by (1-proportion) divided by the total number of participants.

REFERENCES

1. Hamashima C. Cancer screening guidelines and policy making: 15 years of experience in cancer screening guideline development in Japan. *Jpn J Clin Oncol*. 2018;48:278-286.
2. Ministry of Health Labour and Welfare. *Specific Health Checkups and Specific Health Guidance: Annual Health, Labour and Welfare Report 2008-2009.*; 2009. https://www.mhlw.go.jp/english/wp/wp-hw3/dl/2-007.pdf. Accessed 05.08.2024

eTable 1. Validity indicators of self-reported history of cancer screenings and health checkups according to different scenarios using data in the 2017–2019 survey

	Number of participants					Participation rate, % (95% CI)	Sensitivity (95% CI)	Specificity (95% CI)	Concordance rate (95% CI)
	Total	TP	FN	FP	TN				
Lung cancer									
Scenario 1	4,267	1,378	1,201	254	1,434	60.4 (59.0–61.9)	0.53 (0.52–0.55)	0.85 (0.84–0.86)	0.66 (0.64–0.67)
Scenario 2	4,267	1,382	1,394	250	1,241	65.1 (63.6–66.5)	0.50 (0.48–0.51)	0.83 (0.82–0.84)	0.61 (0.60–0.63)
Colorectal cancer									
Scenario 1	4,267	1,415	605	354	1,893	47.3 (45.8–48.8)	0.70 (0.69–0.71)	0.84 (0.83–0.85)	0.78 (0.76–0.79)
Scenario 2	4,267	1,403	684	366	1,814	48.9 (47.4–50.4)	0.67 (0.66–0.69)	0.83 (0.82–0.84)	0.75 (0.74–0.77)
Stomach cancer									
Scenario 1	4,267	618	170	735	2,744	18.5 (17.3–19.6)	0.78 (0.77–0.80)	0.79 (0.78–0.80)	0.79 (0.78–0.80)
Scenario 2	4,267	593	262	760	2,652	20.0 (18.8–21.2)	0.69 (0.68–0.71)	0.78 (0.76–0.79)	0.76 (0.75–0.77)
Scenario 3	4,267	721	332	632	2,582	24.7 (23.4–26.0)	0.68 (0.67–0.70)	0.80 (0.79–0.82)	0.77 (0.76–0.79)
Scenario 4	4,267	722	379	631	2,535	25.8 (24.5–27.1)	0.66 (0.64–0.67)	0.80 (0.79–0.81)	0.76 (0.75–0.78)
Breast cancer									
Scenario 1	2,394	535	99	328	1,432	26.5 (24.7–28.3)	0.84 (0.83–0.86)	0.81 (0.80–0.83)	0.82 (0.81–0.84)
Scenario 2	2,394	519	100	344	1,431	25.9 (24.1–27.6)	0.84 (0.82–0.85)	0.81 (0.79–0.82)	0.81 (0.80–0.83)
Scenario 3	2,394	573	149	290	1,382	30.2 (28.3–32.0)	0.79 (0.78–0.81)	0.83 (0.81–0.84)	0.82 (0.80–0.83)
Scenario 4	2,394	573	141	290	1,390	29.8 (28.0–31.7)	0.80 (0.79–0.82)	0.83 (0.81–0.84)	0.82 (0.80–0.84)
Cervical cancer									
Scenario 1	2,394	610	104	163	1,517	29.8 (28.0–31.7)	0.85 (0.84–0.87)	0.90 (0.89–0.91)	0.89 (0.88–0.90)
Scenario 2	2,394	594	103	179	1,518	29.1 (27.3–30.9)	0.85 (0.84–0.87)	0.89 (0.88–0.91)	0.88 (0.87–0.90)

Scenario 3	2,394	652	178	121	1,443	34.7 (32.8–36.6)	0.79 (0.77–0.80)	0.92 (0.91–0.93)	0.88 (0.86–0.89)
Scenario 4	2,394	653	168	120	1,453	34.3 (32.4–36.2)	0.80 (0.78–0.81)	0.92 (0.91–0.93)	0.88 (0.87–0.89)
Health checkups									
Scenario 1	4,267	2,381	369	547	970	64.4 (63.0–65.9)	0.87 (0.86–0.88)	0.64 (0.63–0.65)	0.79 (0.77–0.80)
Scenario 2	4,267	2,415	555	513	784	69.6 (68.2–71.0)	0.81 (0.80–0.82)	0.60 (0.59–0.62)	0.75 (0.74–0.76)

CI, confidence interval; FN, false negative; FP, false positive; TN, true negative; TP, true positive.

Scenario 1 is a comparison of the responses to the questionnaire with the screening records in the last fiscal year (original meaning).

Scenario 2 is a comparison of the responses to the questionnaire with the screening records within 12 months before the responding month (interpretation error of target period).

Scenario 3 is a comparison of the responses to the questionnaire with the screening records in the last two fiscal years (recall error due to recommendation for screening interval).

Scenario 4 is a comparison of the responses to the questionnaire with the screening records within 24 months before the responding month (a combination of interpretation and recall errors).
